# The Association Between Long-Term Corticosteroids Use and Dental Caries: A Systematic Review

**DOI:** 10.7759/cureus.44600

**Published:** 2023-09-03

**Authors:** Basil M Jan, Mohammed A Khayat, Anaan I Bushnag, Abdullah I Zahid, Abdulaziz S Alkarim, Mohammed T Alshehri, Faisal M Almasoudi, Mohammed Zahran, Soulafa A Almazrooa, Hani H Mawardi

**Affiliations:** 1 Orthodontics, Jacksonville University, Jacksonville, USA; 2 General Dentistry, Ministry of Health, Jeddah, SAU; 3 General Dentistry, Ministry of Health, Turaif, SAU; 4 General Dentistry, King Abdulaziz University, Jeddah, SAU; 5 Prosthodontics, King Abdulaziz University, Jeddah, SAU; 6 Oral Diagnostic Sciences, King Abdulaziz University, Jeddah, SAU

**Keywords:** oral cavity, dmft, long-term, corticosteroids, dental caries

## Abstract

Corticosteroids (CSs) are a group of medications prescribed regularly to treat a wide range of inflammatory and immune-related conditions with great benefit. The impact of long-term use of CSs on the oral cavity has been reported before, including increased risk of periodontal disease and dental caries. Thus, the aim of this study is to evaluate the prevalence of dental caries in patients using CSs. A literature review was completed using PubMed and Cochrane search engines. The search was based on questions related to adults and children (P); corticosteroids (I); no corticosteroids (C); and dental caries (O) (PICO questions) using the keywords “steroids” and “caries” with all relevant variations and MeSH terms. Decay missing filling tooth/decay missing filling surface (DMFT/DMFS) scores were selected as parameters to assess the effects of CSs on caries prevalence. Data was extracted and analyzed for comparisons. The search yielded 1,206 articles from January 2001 to January 2023, of which 21 papers were eligible for analysis. Overall, 14 studies reported an increase in caries with CSs use. However, seven studies failed to report an association of caries prevalence with CSs use. Current evidence supports the correlation between increased risk of caries with chronic CSs use, specifically for inhaler formulation. Future studies with randomized controlled clinical studies are warranted to confirm these findings.

## Introduction and background

Corticosteroids (CSs) are a common group of medications prescribed regularly by physicians for decades to treat a wide range of diseases including inflammatory conditions, allergic reactions, and immune-mediated disorders [1]. The proposed mechanism of action includes the inhibition of inflammatory mediators' synthesis to decrease or prevent an inflammatory event [2]. The routes of administration for CSs vary and include topical, oral/systemic, or injectable application. Historically, the benefits of CSs have been documented in the literature, which helped suffering patients often with life-threatening conditions. However, long-term use of CSs has been linked to a long list of toxicities and adverse events including, but not limited to, adrenal suppression, weight gain, osteoporosis, and increased serum glucose and blood pressure levels [1].

The impact of long-term use of CSs on the oral cavity has been reported before and includes an increased risk of periodontal disease and dental caries, in addition to changes in the properties and function of saliva. These effects have been categorized based on CSs administration routes, including topical formulation, with variable intensities. For instance, the median decayed, missing, and filled teeth (DMFT) score was found to be twofold higher in asthmatic patients on CSs inhalers compared to non-CSs users [3]. In addition, the levels of oral cariogenic bacteria were higher in asthmatic patients on CSs as reported in the literature. Combined with an increase in saliva acidity and decreased flow, these changes may significantly increase the risk for dental caries [4-6]. However, no consensus exists today on the best approach to manage dental patients about to start or currently on CSs therapy.

In order to better understand the effect of CSs on oral health, the aim of this systematic review was to evaluate the risk and prevalence of dental caries in patients with long-term CSs use. We believe the outcome of this review will help to develop national preventive programs and management protocols for this category of patients. 

## Review

Materials & methods

PubMed and Cochrane search engines were used to conduct electronic searches from January 2001 to January 2023. The process used pre-determined questions related to adults and children (P); corticosteroids (I); no corticosteroids (C); and dental caries (O) (PICO questions) using the keywords “steroids” and “caries”, “tooth decay” or ”carious lesion” with all relevant variations and MeSH terms. The most common medical conditions requiring long-term use of CSs were also included in the search process such as “vasculitis”, “Sjogren’s syndrome”, “epidermolysis”, “ulcerative colitis”, “Crohn’s disease”, “systemic lupus erythematosus”, “benign mucous membrane pemphigoid”, “pemphigus vulgaris”, and “asthma”. A citation search was conducted on relevant systematic reviews and meta-analyses to identify eligible studies. Inclusive criteria: studies written in the English language involving human subjects using long-term (> three months) topical and systemic CSs on a regular basis, randomized clinical trials, retrospective, cross-sectional, cohort, and case-control study designs. Exclusion criteria: studies on short-term use of CSs and/or irrigation or local injections with CSs as well as case reports and case series. Out of 1,206 articles from January 2001 to January 2023, 21 articles were eligible for analysis. For the exclusions process, co-authors were split into groups of two authors assigned to review 241 articles. First, included studies were excluded by title then by abstract, and finally by full text. Whenever there was a conflict about a study's eligibility, a group meeting took place to discuss and decide to either include or exclude any particular article. We will consider using software in future studies.

The study flaws/weaknesses: 1) a negative control group for comparing outcomes was used in most of the included studies; 2) most of the included studies failed to control for potential confounding factors such as oral hygiene status, study population, status of regional water supply fluoridation and dietary habits; 3) the duration, specific CSs agent, and frequency and/or dosage of used CSs were not specified in several studies; 4) there was a heterogenicity in parameters used to assess for correlation outcome such as DMFS, mean DMFS and median DMFS which may have affected the interpretation of association data and accounted for outcome heterogenicity. Based on the findings from 14 articles (six case-control studies, six cross-sectional studies, and two cohort studies), there could be a correlation between chronic CSs inhaler use and increased risk of dental caries.

Prior to starting the search process, DMFT/ decay missing filling surface (DMFS) scores and decayed, missing, filled primary teeth and surfaces (dmft/dmfs) were selected as the main parameters to assess the effects of CSs on dental caries prevalence as defined in the literature [7]. The literature search was conducted by seven co-investigators independently (BJ, MK, AZ, AA, MA, AB, FA) in which studies were reviewed starting with titles, abstracts, and finally by full-text. Each step was completed by study co-investigators in pairs. Using the preferred reporting items for systematic reviews and meta-analysis (PRISMA) checklist, the assessment of bias for individuals and across studies was carried over at the study level. In addition, the methodological quality of each study was assessed for flaws in design using the corresponding strengthening the reporting of observational studies in epidemiology (STROBE) checklist in accordance with the STROBE guidelines [8]. Eligible studies were reviewed in group meetings to reach a unanimous agreement. Data was extracted from eligible studies and analyzed for comparisons and significance. 

Results

**Figure 1:**
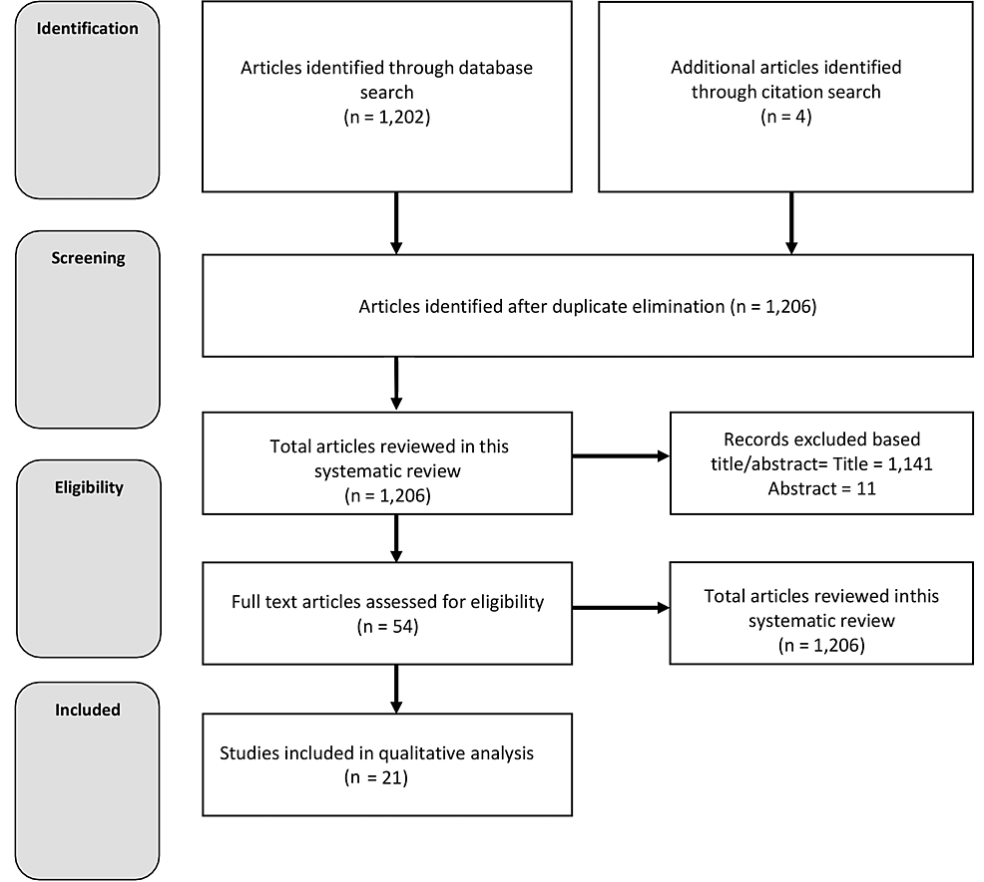
Flow-chart of article search outcome and exclusion process following preferred reporting items for systematic reviews and meta-analysis (PRISMA).

The primary search yielded 1,206 articles, of which 21 articles were eligible for analysis (published from 2001 to 2023) and included in this systematic review (Figure 1). Overall, there were nine case-control studies, nine cross-sectional studies, and three cohort studies. Complete details of included studies are listed in Tables 1, 2.

**Table 1:** All studies supporting a potential role for chronic CS use to increase the risk of dental caries. CSs: Corticosteroids; DMFT/S=decayed, missing, filled permanent teeth/surfaces; dmft/s=decayed, missing, filled primary teeth/surfaces; D1MFS=non-cavitated and decayed, missing, and filled surfaces; D2MFS=cavitated decayed, missing and filled surfaces; MT= missing teeth (due to caries);* = This article does not mention specific types and/or doses and/or duration of CSs in all or some aspects of the study.

Author (year)	Study type	Study population (N)	Details of CSs used	Risk/prevalence of dental caries	Methodological quality assessment score
Samec et al. [9]	Cohort	A total of 586 aged from 2-17 years old were included in the study: 138 asthmatic children 140 Non-asthmatic siblings (controls) Additional 308 Asthmatic children were further included in the second part of the study for additional investigations study population was divided into three age groups 1st group: 2- to 6-year-old children (n = 72). 2nd group: 7- to 12-year- old children (n = 153). 3rd group: 13- to 17-year-old.	All asthmatic children included used Inhaled glucocorticoid daily for at least 1 year (mean length of anti-asthmatic medication use was 5.46) Specific doses and medications were not reported	Asthmatic children had significantly lower prevalence of sound tooth surfaces in the first and third age groups with a mean of 82.17 and 134.86 compared with their non-asthmatic siblings with a mean of 91.79 and 142.60 respectively (p < 0.01, p < 0.05) Asthmatic children had significantly (p < 0.05) higher mean d12fs in year 2008 and in 2011 with a mean of 10.94 and 8.88 respectively, compared to non-asthmatic siblings on their primary teeth with a mean of 4.09 and 2.91, as well as significantly higher mean D12MFS on their permanent teeth (p < 0.01)	18/22
Hassanpour et al.* [10]	Case-control	140 children (aged 3-12 years-old): - 70 asthmatic - 70 healthy	70 patients received inhaled CS for ≥ 1 year (mean duration 1.90 years)	- Mean DMFT was 0.71 in asthmatics compared to 0.48 in healthy group (p=0.002) - Mean dmft score was 3.79 in asthmatics compared to 2.32 in healthy group (p=0.001) - Mean decayed, missing, and filled teeth were 2.41, 1.13, 0.96 in asthmatics compared to 1.62, 0.67, 0.51 in healthy group (p=0.001, p=0.009, p=0.014)	10/22
Wu et al.* [11]	Retrospective cohort	9190 children (aged 0-9 years old): - 4601 asthmatic - 4589 healthy	- 584 patients received inhaled CSs only. - 1565 patients received a combination of a bronchodilator and inhaled CSs. - 2452 patients received bronchodilators only	Caries prevalence: -asthmatics using CSs (91.3%) -bronchodilators (92.9%) -healthy children (85.2%) -combined CSs with bronchodilator users (84.9%) (p<0.001) - asthmatics taking quick-relief agents (short-acting β2 agonists and short-acting muscarinic receptor agonists) (92.8%) - asthmatics taking long- acting β2 agonists and long-acting muscarinic receptor agonists and CSs (89.3%) - asthmatics taking combination treatment with or without CSs (85.3%) (p<0.001)	18/22
Raj et al.* [12]	Cross-sectional	340 adults (aged 20-45 years-old): - 170 COPD patients on medication for ≥6 months - 170 healthy	- 4 patients received CSs. - 147 patients received CSs and long-acting β agonists - 8 patients received an anticholinergic agent, CSs, and long-acting β agonists - 2 patients received CSs, long and short-acting β agonists - 2 patients received short-acting β agonists Medication frequency: -7 patients took medication once daily - 151 patients took medication twice daily -12 patient took medication 3 times a day	- mean DMFT score was 8.27 among COPD patients; 6.35 for controls (p≤0.002) - mean DMFT score was 10.77 in patients receiving medication for >5 years; 7.62 in those receiving medication for ≤5 years (p≤0.005)	18/22
Chellaih et al.* [13]	Cross-sectional	110 children (aged 6-14 years-old): - 55 asthmatic - 55 healthy	55 subjects received daily inhaled CSs (agent not specified) combined with inhaled β2 agonist for ≥2 years	- Mean DMFT for asthmatics was 4.53; non-asthmatics was 1.51 (p<0.001) - Subjects using inhaled CSs were 6.26 times more likely to develop dental caries (95% CI 2.6–14.9)	10/22
Boskabady et al.* [14]	Cross-sectional	80 adults (aged 20-30 years-old): - 40 asthmatic - 40 healthy	40 patients received inhaled beclamethasone dipropionate (600-1600 µg), fluticasone propionate (500 µg), salmeterol (50 µg) or salbutamol (400 µg as needed).	- higher MT and DMFT in asthmatics compared to non-asthmatics (p<0.005) - No significant difference in dental caries score, medication dose or inhalation use technique between different severities of asthma - No significant correlation between dental caries score and dose of medication or inhalation use technique score	13/22
Samec et al.* [15]	Cross-sectional	440 subjects (aged 2-17 years-old): - 220 asthmatic - 220 healthy	All asthmatic patients received CSs daily; and a bronchodilator when needed: - 173 patients received inhaled CSs - 35 patients received dry-powder inhaled CSs Medication duration: - 35 patients used the medication for 1 year - 159 patients used the medication for 2-8 years - 26 patients used the medication for 9-16 years	- Asthmatics aged 2-6 showed higher d1, d2, d2fs, and d12fs and less caries-free children compared to non-asthmatics (p<0.01 and p<0.05) - Asthmatics aged 7-12 showed higher d1, d2, d2fs, d12fs, D1, D2, F, D1MFS, D2MFS, and D12MFS compared to non-asthmatics (p<0.01) - High CSs dose, frequency, and duration of medication use were associated with high D12MFS (p>0.01)	16/22
Santos et al.*[3]	Cross-Sectional	80 subjects (aged 10-18 years-old): - 40 asthmatic - 40 healthy	Patients received inhaled CSs for ≥3 months and inhaled beta-2 agonists for less than once a week	- The median DMFT and DMFS was 3 and 4 for asthmatics; 1.5 and 1.5 for non-asthmatics (p=0.00; p=0.007)	12/22
Botelho et al.* [16]	Cross-sectional	160 children (aged 3-15 years-old): -80 asthmatic -80 healthy	- 13 patients received CSs only - 15 patients received CSs and bronchodilators - 52 patients received bronchodilators only Medication frequency (including bronchodilator-only group): - 41 patients used the medication for acute crises or irregularly - 34 patients used the medication regularly Medication duration (including bronchodilator-only group): - 27 patients used the for <2 years - 22 patients used the medication for 2-4 years - 31 patients used the medication for >4 years	- No significant difference in overall caries prevalence between asthmatics and non-asthmatics (p>0.05) - Accounting for age, asthmatics aged 11-15 years had DMFT 2.11; non-asthmatics had DMFT 1.05 (p=0.024) - No significant correlations were observed between the type of medication, frequency and method of consumption and caries prevalence	15/22
Stensson et al. [17]	Cross-sectional	40 adults (aged 18-24 years-old): - 20 asthmatic - 20 healthy	Medication type: - 8 patients received fluticasone with a mean dose of 240 (77.1) µg/day and salmeterol - 12 patients received budesonide with a mean dose of 320 (71.5) µg/day and formoterol Medication frequency: - 16 patients used the medication twice a day - 1 patient used the medication >2 times a day - 4 patients used the medication regularly Mean overall duration of asthma medication use was 13.5 years.	- Initial mean caries and approximal carious lesions prevalence were higher in the asthma group (6 and 4.1) compared to the control group (1.3 and 0.7) (p=0.02) (p=0.01) - No significant difference in mean DFS between asthmatics who experienced the disease before age 5 years (n=13) compared to those at age ≥5 years (n=7) - Mean DFS for asthmatics with asthma for <9 years (n=4) was 4.2; for asthmatics with a duration of asthma of >9 years (n=16) was 9.7 for (p=NS) - No difference in mean DFS between both asthma medication subgroups were noted	15/22
Mehta et al.* [18]	Case-control	160 subjects (aged 11-25 years-old): - 80 asthmatic - 80 healthy	All asthmatic patients received CSs combined with β2-agonists for ≥ 6 months (with at least one of the medications via inhalation)	- Mean DMFT and DMFS scores were 3.73±2.03 and 6.38±4.66 for asthmatics; 1.30±0.97 and 2.08±2.20 for the healthy group (p<0.001) - Mean DMFT and DMFS for patients receiving fluticasone proprionate and salmeterol were 4.26±1.93 and 7.21±4.70, beclomethasone diproprionate were 3.67±1.61 and 6.17±3.66, and budesonide were 3.53±2.16 and 6.10±4.90 (p=0.416, p=0.675) - A positive correlation was observed between duration of asthma and both, DMFT and DMFS (p = 0.016, p = 0.021)	11/22
Shashikiran et al.* [19]	Case-control	211 children (aged 6-14 years-old): - 105 asthmatic - 106 healthy	- 35 patients received beclamethasone inhaler - 35 patients received salbutamol inhaler - 35 patients received salbutamol tablets	- Salbutamol inhaler group had higher caries parameters (dft, dfs, DMFT, DMFS) compared to control group (p<0.05) - CSs group had mean dft of 0.43 and dfs of 0.54, the control group had 0.14 and 0.14 (p<0.05) - No significant differences were noted between mean DMFT and DMFS for CSs group (0.22 and 0.42) and control group (0.40 and 0.43) (p>0.05)	12/22
Ersin et al.* [20]	Cross-sectional	206 subjects (aged 6-19 years-old): - 106 asthmatic - 100 healthy	- 38 patients received inhaled CSs and β2-agonists - 55 patients received inhaled CSs and β2-agonists with an anti-inflammatory agent or leukotriene antagonists - 13 patients received inhaled β2-agonists only - Mean overall duration of drug administration was 1.92 years	- Asthmatics aged 6-10 years had mean dfs of 9.2 and DMFS of 3.3; healthy group had 5.7 and 1.5 (p<0.05) - Asthmatics aged 11-19 years had mean DMFS of 4.1; healthy group had 3.8 (p<0.05)	14/22
Milano et al.*[21]	Cross-sectional	156 children: - 104 asthmatic in primary dentition (aged 31-141 months). - 52 asthmatic in mixed dentition (aged 58-141 months).	Medication type: - 116 patients received albuterol inhaler only (76 in primary and 40 in mixed dentition) - 20 patients received albuterol and cromolyn aerosol inhaler (16 in primary and 4 in mixed dentition) - 20 patients received prednisone in combination with albuterol and cromolyn aerosol inhalar (12 in primary and 8 in mixed dentition) Medication frequency: - 24 patients used the medication once a day - 60 patients used the medication twice a day - 44 patients used the medication 3 times a day - 28 patients used the medication ≥4 times a day Medication duration: - 12 patients used the medication for <1 year - 36 patients used the medication for 1-2 years - 20 patients used the medication for 2-3 years - 24 patients used the medication for 3-4 years - 12 patients used the medication for 4-5 years - 52 patients used the medication for >5 years	- Asthmatics taking medication >2 times a day were more likely to experience caries in primary dentition (p<0.05); less likely to have caries in mixed dentition - Asthmatics taking medication >2 times a day were more likely to experience caries in mixed dentition	18/22

**Table 2:** All studies reporting no impact of chronic CS use on prevalence of dental caries. CSs=corticosteroids; DMFT/S=decayed, missing, filled permanent teeth/surfaces; dmft/s=decayed, missing, filled primary teeth/surfaces; D1-2=caries without a cavity in permanent teeth; d1-2=caries without a cavity in primary teeth; D3-6=caries with a cavity in permanent teeth;d3-6=caries with a cavity in primary teeth.* = This article does not mention specific types and/or doses and/or duration of CSs in all or some aspects of the study.

Author (year)	Study type	Study population (N)	Details of CS use	Risk/prevalence of dental caries	Methodological quality assessment score
Doğan et al. [22]	Cross -sectional	-115 children with asthma -Mean age 54 months old (range 42-66)	-The duration of using inhaled CSs was 24 months. -74.8 % of the children included used Flixotide propionate while 25.2% used budesonide+lactose	-The mean dmf/DMF scores for teeth and surfaces were: dmft=2.69±3.58, dmfs=4.97±8.27, DMFT=0.28±0.58 DMFS=0.28±0.58 -No significant differences were found in terms of dental caries (dmft/DMFT scores)	14/22
Cerrate et al.* [23]	Cross-sectional	184 children (aged 3-13 years-old: - 92 asthmatics - 92 healthy	- 80 patients received budesonide and salbutamol - 12 patients received fluticasone and salmeterol Medication frequency: - 89 patients used CSs 2 puffs/day - 3 patients used CSs >2 puffs/day Medication duration: - 12 patients used the medication for 1-2 years - 32 patients used the medication for 2-4 years - 48 patients used the medication for >4 years	- A significant relationship was observed between treatment duration with inhalers and rate of dental caries (p=0.04) - DMFT index for patients taking medication for 1-2 years was 1.91, 3.46 for 2-4 years, and 4.27 for >4 years (p=0.04) - No significant differences were observed in relation to caries prevalence between asthmatics (28.3%) and healthy (34.2%) (p=0.094) -No significant differences were observed in relation to DMFT index between asthmatics (3.98) and healthy (4.73) (p=0.08)	19/22
Rezende et al.* [24]	Cross-sectional	228 children (aged 6-12 years-old): - 112 asthmatics - 116 non-asthmatics	- 68 patients received salbutamol with beclomethasone or budesonide with systemic CSs - 37 patients received salbutamol only - 7 asthmatic patients received no medication	- 25.2% of asthmatics on a combination of medications including CSs had dental caries compared; 53.7% in no medication group (p=0.053) - Salbutamol group had a statistically significant increase in dental caries compared to the no medications group	16/22
Bahrololoomi et al. [25]	Cross-sectional	93 children (aged 6-12 years-old): - 46 asthmatics - 47 non-asthmatics	Asthmatics patients were treated for a duration ranging between 6 months to 5 years using one of the following combinations: - budesonide/formoterol fumarate (160 g/4.5g and 320g/9g) - salmeterol/fluticasone propionate (25g/125g and 25g/250 mg) - beclomethasone propionate monohydrate (50 mg and 100 mg) - fluticasone propionate (125 mg and 250 mg)	- No significant difference in dmft score was observed between asthmatics and non-asthmatics (p=0.26) - Mean numbers of decayed, missing, filled, and dmf in asthmatics were 3.34, 0.32, 0.78, and 4.15 compared to 3.78, 0.27, 1.19, and 5.25 in controls (p>0.05) - Variables related to asthma medication including type of inhaler (p=0.539) and daily frequency of inhaler use (p=0.906) had no significant effect on dmft score	16/22
Kilinc et al., [26]	Cohort	102 children (aged 4-16 years-old): - 51 asthmatics - 51 non-asthmatics	- 45 children received budesonide (400 µg) for 1 year - 6 children received budesonide (200 µg) for 1 year - 9 (out of 51) children received additional montelukast tablets at follow up	At baseline (before starting asthma medication): - Decayed teeth for asthmatics aged 4-6 years was 9.8%; 13.7% for non-asthmatics - Decayed teeth for asthmatics aged between 7-11 and 12-16 years was 41.18% and 49% for; 56.86% and 29.41% for non-asthmatics - The rate of decayed teeth in asthmatics was 1.032-1.38 times compared to non-asthmatics 6-months post-therapy: - 3 new D3-6+d3-6 and 1 D1-2 dental caries were found in asthmatics, compared to 1 D1-2 and D3-6 caries in non-asthmatics - The rates of new dental caries were similar in asthmatics and non-asthmatics (0.021-1.50 times) - Children in both groups had extraction events related to caries during this period. 1-year post-therapy: - No new D3-6 caries in asthmatics compare rd to 1 in a child of the non-asthmatics - 1 D1-2 caries was observed in an asthmatic child; none in non-asthmatics	18/22
Eloot et al. [27]	Cross-sectional	140 asthmatic children (aged 3-17 years-old)	Medication type: - 33.3% of patients received β2-mimetics daily and <400 mcg/day inhaled CS - 47.3% received >400 mcg/day or >1000 mcg/day of inhaled CS Medication duration: - 8% of patients used medication for <2 years - 31.4% of patients used medication for 2-5 years - 48.9% of patients used medication for 5-10 years - 11.7% of patients used medication for >10 years	- No significant differences were reported between the dmf-values of asthmatic groups with different exposure to medication (p>0.05)	14/22
Wogelius et al. [28]	Cohort	785 children (aged 3-7 years-old) - 578 asthmatic receiving medication - 207 receiving no medication 4920 children (aged 3-7 years-old) - 283 received both β2-agonists and CSs inhalers between 3-5 years - 295 received both β2-agonists and CSs inhalers between 5-7 years - 3157 did not receive any medication	- 169 subjects received β2-agonists and CSs inhalers at ages 3-5 years old - 295 subjects received β2-agonists and CSs inhalers at ages 5-7 years old	- Slightly more asthmatics with caries-free primary canines and molars aged 7 years old than control group - Slightly more dmfs ranging from 1 to 4 in asthma medication group than control group - More permanent teeth, caries-free in control group compared to study group - Relative risk of caries in permanent teeth of asthmatics receiving medication was 1.62 for ages 3-7 years old (p=0.04) - No significant relative risk for asthma medication in relation to caries of primary canine and molar (p>0.05)	17/22

Increase in dental caries with CSs use

In total, 14 articles reported a positive correlation between CSs use and dental caries. For case-control studies, Hassanpour et al. conducted a study comparing 70 asthmatic children receiving inhaled CSs for a year or longer to 70 age-matched healthy children and found significantly higher mean DMFT, mean dmft, in addition to higher mean decayed, missing, and filled teeth in the asthmatic group [10]. Chellaih et al. conducted a study comparing 55 asthmatic patients receiving daily inhaled CSs and bronchodilators for two years or more to 55 healthy subjects and reported significantly higher mean DMFT in the asthmatic group with a risk of 6.26 times to develop caries [13]. Stensson et al. compared 20 asthmatic patients (eight receiving fluticasone with a mean dose of 240 µg/day and salmeterol, and 12 receiving budesonide with a mean dose of 320 µg/day and formoterol) to 20 healthy subjects. There was an increase in the prevalence of caries in asthmatic patients with no significant differences in mean decayed and filled surfaces (DFS) in relation to age, type of asthma medications, and doses [17]. Mehta et al. conducted a study in which 80 asthmatic patients (aged 11-25 years old) receiving either CSs or β2-agonists inhalers for six months or longer were compared to 80 age-matched healthy controls. Statistically significantly higher mean DMFT and DMFS in asthmatics were reported. However, no significant differences in mean DMFT and DMFS were reported between both groups based on inhaled agents (fluticasone propionate, salmeterol, beclomethasone propionate, or budesonide) [18]. Shashikiran et al. compared 105 asthmatic patients (35 receiving inhaled beclomethasone for one year, 35 receiving bronchodilators, and 35 receiving salbutamol tablets) to 105 healthy subjects, and reported that subjects receiving CSs inhalers had significantly higher dft and dfs compared to the control group. However, no significant differences in DMFT and DMFS were found between CS users and healthy subjects. However, the bronchodilator group had significantly higher DMFT and DMFS scores compared to healthy subjects [19]. Ersin et al. compared 106 asthmatic patients (38 receiving inhaled CSs and bronchodilators, 55 receiving inhaled CSs, bronchodilators with anti-inflammatory agents or leukotriene antagonists, and 13 receiving only bronchodilators) to 100 healthy subjects. They found that asthmatic subjects aged between six and 10 years had significantly higher mean dfs and DMFS. However, no difference in mean DMFS was noted in the 11-19 years old groups [20].

Several cross-sectional studies were conducted to assess the relation between chronic CSs use and dental caries. For instance, Raj et al. conducted a study that included 161 chronic obstructive pulmonary disease (COPD) patients receiving either CSs alone (N=4), CSs and bronchodilators (N=147), CSs, bronchodilators and an anticholinergic agent (N=8), or bronchodilators only (N=2). Compared to healthy patients, COPD patients had an increase in DMFT, specifically for those on active CSs for five or more years [12]. Boskabady et al. included 40 asthmatic patients who were actively using beclomethasone dipropionate (600-1600 µg) or fluticasone propionate (500 µg) inhalers, with bronchodilators when needed, and observed an increase in DMFT and missing teeth due to caries compared to 40 healthy subjects. In addition, no significant correlation between caries risk and medication dose or usage technique was noted [14]. A study by Samec et al. included 220 asthmatic patients actively using CSs inhalers with bronchodilators as needed and observed an increase in early decayed and filled surfaces in patients aged between two to 12 years old compared to 220 matched-healthy subjects. No statistically significant impacts of CSs dose, frequency, or duration on caries risk were reported [15]. Santos et al. conducted a study on 40 asthmatic patients receiving CSs inhalers and observed increased median DMFT and DMFS compared to 40 healthy subjects [3]. Botelho et al. conducted a study and recruited 80 asthmatic patients, 13 of whom have been receiving CSs alone, 15 receiving CSs and bronchodilators combined, and 52 receiving bronchodilators alone. Compared to 80 healthy subjects, no significant difference in caries incidence was noted among asthmatic patients in general. However, a higher caries prevalence was observed in asthmatic patients aged between 11-15 years old when stratified resulting in an increase in DMFT compared to healthy subjects. No role for medication type, frequency, or method of consumption in caries risk was reported [16]. Milano et al. conducted a study with 20 asthmatic patients receiving prednisone in addition to albuterol and cromolyn aerosol-inhalation and reported an increased risk for primary and mixed dentition caries in patients on this medication more than two times a day [21].

Samec et al. conducted a cohort study comparing 138 asthmatic subjects actively using inhaled CSs for at least one year (divided into a group of 72 children aged two to six years old, 153 children aged seven to 12 years old, and a group of 13-17-year-old subjects) to 140 non-asthmatic siblings [9]. Asthmatic subjects in the first and third groups were found to have a significantly lower prevalence of sound teeth surfaces compared to the control group, and asthmatic children had significantly higher d12fs and D12MFS than their non-asthmatic siblings. A retrospective cohort study compared 9190 asthmatic subjects (584 patients were receiving inhaled CSs, 1565 receiving CSs inhalers combined with a bronchodilator, and 2452 receiving bronchodilators only) to 4589 healthy subjects [11]. A statistically significant increase in caries prevalence among CSs users and fewer caries-free children were reported. In addition, subjects receiving bronchodilators had a significantly higher prevalence of caries than CSs users and less caries-free children.

No increase in dental caries with CSs use

Several studies reported no relation between the risk of dental caries and chronic CSs use. For case-control studies, Cerrate et al. compared 92 asthmatic patients receiving CSs (inhaler or nasal spray) in combination with bronchodilators to 92 healthy subjects and reported an overall increased rate of caries and DMFT in association with longer duration of CSs inhaler use [23]. However, no significant differences in caries prevalence and DMFT were noted between both groups. Another study compared 46 asthmatic patients receiving different CSs inhalers with or without bronchodilators, including budesonide/formoterol fumarate (160 g/4.5g and 320g/9g), salmeterol/fluticasone propionate (25g/125g and 25g/250 mg), beclomethasone propionate monohydrate (50 mg and 100 mg), and fluticasone propionate (125 mg and 250 mg) to 47 healthy subjects and reported no significant difference in dmft between both groups [25]. In addition, the type of inhaler and frequency of medication used had no significant effect on the overall outcome. Kilinc et al. compared 51 asthmatic patients receiving CSs inhalers for one year to 51 healthy subjects and reported no difference in the rate of caries incidence between both groups [26]. 

For cross-sectional studies, Doğan et al. conducted a study with 115 asthmatic children aged 44-66 months-old using inhaled CSs for 24 months, in which 74.8% were on flixotide propionate and 25.2% on budesonide combined with lactose. At the end of the study, no significant differences in dmft/DMFT scores were noted [22]. Rezende et al. conducted a study comparing 112 asthmatic patients (68 subjects on long-term bronchodilators and beclomethasone inhalers or budesonide inhalers, as well as systemic CSs, 37 receiving bronchodilators only, and seven, were not on any medication) to 116 healthy subjects [24]. The study reported no significant difference in caries risk between subjects receiving CSs with bronchodilators and those receiving no medication from both groups. However, an increase in dental caries risk was noted in the bronchodilator group when compared to those receiving no medication. Eloot et al. also reported no relation between dmf and exposure length to medication in 140 asthmatic children aged three to 17 years old and receiving bronchodilators daily combined with variable doses of CSs inhalers [27].

A cohort observational study examined 785 subjects, of which 578 were asthmatics receiving inhaled bronchodilators in addition to CSs and 207 were healthy children. At the end of the study, there were more asthmatic patients with caries-free primary molars and canines compared to medication-free subjects [28]. However, the healthy group had less dmfs 1-4 values, and more permanent teeth with no caries. No significant relative risk was noted in inhaled CSs and bronchodilator users for developing primary canine and molar caries. The reported relative risk was 1.45 for developing new carious lesions in permanent teeth of patients receiving inhaled CSs and bronchodilators between five to seven years old and increased to 1.62 when including patients from ages three to seven years old.

Discussion

CSs are a group of synthetic, anti-inflammatory agents commonly prescribed for a variety of immune-related diseases such as asthma, COPD, and systemic lupus erythematosus [29]. Over the past decades, delivery of CSs via different routes has helped millions of sick people and improved the overall quality of life with a reasonable safety profile for short-term use. However, chronic use of CSs has been associated with systemic toxicities such as hypertension, diabetes, osteoporosis, and many more [30]. Moreover, the impact of CSs use on oral health is unclear and may include a decrease in salivary flow, lower pH, higher bacterial levels of Streptococcus mutans (SM) and Lactobacilli (LB) as well as increased risk of dental caries [31]. Hence, this systematic review aimed to assess the relationship between chronic CSs use and the risk of dental caries.

Dental caries is the most common, multifactorial, chronic oral disease of humans, specifically for children above the age of six and adolescents [32, 33]. Its pathogenesis is driven by a cariogenic bacteria-supported biofilm to maintain a low pH and initiate sub-surface demineralization, followed by prolonged periods of repeated demineralization and remineralization activities leading to net demineralization outcome [34]. It is estimated that dental caries of permanent teeth have affected 2.3 billion people worldwide and 530 million children with primary teeth caries in 2017 [35]. Several risk factors have been linked to dental caries including poor diet, lower socioeconomic status, and underlying genetic susceptibility of tooth morphology [36]. However, the exact role of long-term use of CSs on increased risk of dental caries has yet to be investigated.

Based on the available literature, several underlying mechanisms have been proposed to explain the potential impact of long-term use of CSs on dental caries prevalence. For instance, the decrease in salivary pH level induced by CS inhalers may promote demineralization and facilitate the development of dental caries [37]. Furthermore, lactose (fermentable carbohydrate) can be an inactive component of CSs inhalers such as beclometasone, budesonide, flunisolide, and fluticasone and act as a substrate for cariogenic bacteria [38]. While lactose is less cariogenic compared to other carbohydrates, it carries a higher cariogenic activity in the presence of dental plaque [39, 40]. Inhaled CSs have also been reported to increase dental plaque accumulation among adolescents; thus, increasing the risk for the development of caries [3]. Similar to inhalers, systemic CSs have been found to decrease salivary pH and increase salivary viscosity, which both could increase the risk of dental caries [41-43]. As of today, the exact mechanism behind the increased risk of dental caries with chronic CSs use is yet to be determined.

In this systematic review, the available evidence was categorized based on the type of correlation as either an increased risk or no relation of dental caries with CSs chronic use. While 14 articles reported a negative impact of CSs use on the prevalence of dental caries, only seven reported no significant correlation. The quality of these studies varied as well, in which 47% of the included studies had a quality assessment score of >15 out of 22, while the rest had a score of <15 using STROBE statement checklists for quality assessment. As the minimum acceptable quality score for any study to be considered is 10, studies reporting no significant correlation between dental caries risk and long-term CSs use (Table 2) had higher mean of quality assessment scores with a minimum included score of 14. On the contrary, studies reporting an association between dental caries risk and long-term CSs use had scores ranging from 10 to 18 in which five studies scored 13/22 in the total quality assessment score. As such, the level and quality of evidence were too minimal to determine a true correlation. In addition, factors such as duration, specific CSs type, frequency, and/or dosage of used CSs were not accounted for nor specified in several studies. Combined with variation and heterogenicity in outcome parameters, the weight of included studies related to the impact of CSs chronic use on the increase in caries risk is questionable and should be further verified.

Asthma and COPD are common respiratory conditions with a reported prevalence of 358 million and 328 million in 2015, both in which CSs inhaler is commonly prescribed as a standard treatment option [44]. Therefore, most of the subjects recruited in the included studies were from those patients’ categories. In general, CSs inhalers have been associated with potential side effects in the mouth including xerostomia and risk of secondary oral candidal infection [45]. Based on the current data, CSs inhalers were also associated with increased risk of dental caries. Whether the combination of topical with systemic CSs has a synergistic effect was not evaluated in the included studies and has to be further investigated. Based on the current evidence, it is reasonable to consider subjects on long-term topical and/or systemic CSs to be at an increased risk of dental caries. As such, close monitoring with regular dental visits for dental examination, regular fluoride application, and early detection of carious lesions are warranted.

Several studies reported a tendency for higher caries risk via reduction of salivary flow in patients using CSs agents combined with other concomitant medications such as anti-histamines and bronchodilators [5, 46, 47]. Furthermore, combining CSs with anti-asthmatic agents including β2-agonists and anti-histamines has demonstrated an increase in salivary cariogenic bacterial levels including SM and LB [4, 48]. Whether the use of anti-histamines and/or bronchodilators with CSs imposes a synergistic effect and increases the risk for dental caries compared to CSs alone has not been evaluated in any of the included studies. More causality studies are needed to better understand this observation.

The current systematic review has multiple limitations. First, most of the included studies used a negative control group for comparing outcomes. Second, less than half of the included studies controlled for confounding factors such as oral hygiene status, study population, status of regional water supply fluoridation, and dietary habits [12, 14, 15, 17, 20, 27, 49]. Furthermore, the duration, specific CSs agent, and frequency and/or dosage of used CSs were not controlled nor specified in several studies. Fourth, variable parameters were used to assess for correlation outcomes such as DMFS, mean DMFS, and median DMFS which may have affected the interpretation of association data and accounted for outcome heterogenicity.

## Conclusions

The main study question focuses on the possible impact of long-term CSs use on the oral cavity, specifically the risk of dental caries. The study claims that long-term use of CSs may have a detrimental effect on the oral cavity which includes an increased risk of dental caries. The aim of this study was to evaluate the prevalence of dental caries in patients with long-term use of CSs. Based on the available literature, several underlying mechanisms have been proposed to explain the potential impact of long-term use of CSs on dental caries prevalence. For instance, the decrease in salivary pH level induced by CS inhalers may promote demineralization and facilitate the development of dental caries. Furthermore, lactose (fermentable carbohydrate) can be an inactive component of CSs inhalers such as beclometasone, budesonide, flunisolide, and fluticasone and could act as a substrate for cariogenic bacteria. A systematic review was conducted, in which 21 articles were eligible and included in the analysis. In total, 14 studies reported an increase in caries prevalence with chronic CSs use more commonly in CS inhaler users. However, seven studies failed to report an association between caries prevalence and long-term CSs use. The available evidence could support the correlation between chronic CSs use and increased risk of caries which requires focused dental education and continuous special dental care for this group of patients. Considering that this is the first systematic review to be reported in the literature focusing on the relationship between long-term usage of CSs and the risk of dental caries, it is not feasible to compare these results to other reviews. Future randomized controlled clinical studies are warranted to confirm these findings.
